# An Overview of Novel Unconventional Mechanisms of Hematopoietic Development and Regulators of Hematopoiesis – a Roadmap for Future Investigations

**DOI:** 10.1007/s12015-019-09920-4

**Published:** 2019-10-22

**Authors:** Kamila Bujko, Monika Cymer, Mateusz Adamiak, Mariusz Z. Ratajczak

**Affiliations:** 1 Stem Cell Institute at James Graham Brown Cancer Center, University of Louisville, 500 S. Floyd Street, Rm. 107, Louisville, KY 40202 USA; 2 Center for Preclinical Studies and Technology, Department of Regenerative Medicine, Medical University of Warsaw, Warsaw, Poland

**Keywords:** Hematopoietic stem cells, Chemokines, Purinergic signaling, Bioactive phosphosphingolipids, Stem cell trafficking, Sterile inflammation, Hormones, VSELs, Stem cell mobilization

## Abstract

Hematopoietic stem cells (HSCs) are the best-characterized stem cells in adult tissues. Nevertheless, as of today, many open questions remain. First, what is the phenotype of the most primitive “pre-HSC” able to undergo asymmetric divisions during ex vivo expansion that gives rise to HSC for all hemato-lymphopoietic lineages. Next, most routine in vitro assays designed to study HSC specification into hematopoietic progenitor cells (HPCs) for major hematopoietic lineages are based on a limited number of peptide-based growth factors and cytokines, neglecting the involvement of several other regulators that are endowed with hematopoietic activity. Examples include many hormones, such as pituitary gonadotropins, gonadal sex hormones, IGF-1, and thyroid hormones, as well as bioactive phosphosphingolipids and extracellular nucleotides (EXNs). Moreover, in addition to regulation by stromal-derived factor 1 (SDF-1), trafficking of these cells during mobilization or homing after transplantation is also regulated by bioactive phosphosphingolipids, EXNs, and three ancient proteolytic cascades, the complement cascade (ComC), the coagulation cascade (CoA), and the fibrinolytic cascade (FibC). Finally, it has emerged that bone marrow responds by “sterile inflammation” to signals sent from damaged organs and tissues, systemic stress, strenuous exercise, gut microbiota, and the administration of certain drugs. This review will address the involvement of these unconventional regulators and present a broader picture of hematopoiesis.

## Introduction

Hematopoietic stem/progenitor cells (HSPCs) are the best-studied stem cells and have been widely employed in the clinic for 50 years. In addition to bone marrow (BM) as a source of HSPCs [1], cells for transplantations are derived from mobilized peripheral blood (mPB) [2] and umbilical cord blood (UCB) [3]. Several in vitro and in vivo assays have been proposed to study their hematopoietic potential in experimental settings and to use as tools to help in the diagnosis of hematological disorders [4]. Nevertheless, there remain some unanswered questions in this field and some unresolved controversies.

The classical in vitro clonogenic assays for characterizing HSPC commitment to different lineages are based on the application of selected peptide-based hematopoietic growth factors (kit ligand [KL]) and cytokines (granulocyte–macrophage colony stimulating factor [GM-CSF], granulocyte colony-stimulating factor [G-CSF], macrophage colony-stimulating factor [M-CSF], interleukin 3 [IL-3], erythropoietin [Epo], and thrombopoietin [Tpo]) [4]. However, evidence has accumulated that in addition to this potent factors there are several peptide-based and non-peptide-based modulators of hematopoietic development. Examples include pituitary somatotropins (follicle-stimulating hormone [FSH], luteinizing hormone [LH], prolactin [PRL], and growth hormone [GH]) and gonadal sex hormones (estrogens, androgens, and thyroid hormones) [5–9].

A major role in the migration of HSPCs has been assigned to the α-chemokine stromal-derived factor 1 (SDF-1) [10, 11]; however, evidence has accumulated that bioactive phosphosphingolipids (sphingosine-1-phosphate [S1P] and ceramide-1-phosphate [C1P]) [12–14] as well as nucleotides secreted into the extracellular space (e.g., adenosine triphosphate [ATP]) and nucleosides (e.g., adenosine) [15–17] may affect hematopoietic development and postnatal migration of HSPCs.

Finally, the appropriateness of the available in vitro and in vivo animal models for studying hematopoietic development is clouded by several issues. First, we still do not know what is the most primitive stem cell (“pre-HSC”) endowed with hematopoietic potential that resides in postnatal tissues. Such cells should undergo asymmetric divisions, and conclusive evidence for the existence of the most primitive CD34^+^ CD45^+^ lin^−^ HSCs currently proposed is still missing. Moreover, for obvious reasons, murine HSPCs are tested in vivo in long-term engrafting assays and serial transplants in mice, but human HSPCs are tested in vivo in immunodeficient mice, sheep fetuses, and other surrogate models (e.g., humanized mice) to better mimic the human hematopoietic microenvironment [4]. However, these assays are sometimes difficult to perform (e.g., serial transplants with human cells in immunodeficient mice), expensive (e.g., large animal models) and not always easy for interpretation. Useful information about hematopoietic development is gained in the zebrafish model, but since hematopoiesis in zebrafish occurs in the kidney instead of in bone marrow (BM), this otherwise elegant model has also some downsides [18].

Finally, it is clear that major hematopoietic organs are affected by signals derived from the neural system [19, 20], the gut microbiota [21, 22], the redox (reduction and oxidation) potential of the hematopoietic microenvironment [23, 24], and the biological effects from activation of the complement cascade (ComC), the coagulation cascade (Coa), and the fibrinolytic cascade (FibC) [25, 26]. In addition, extracellular microvesicles (ExMVs) [27, 28], which are secreted by cells in BM microenvironment, have emerged as new players in cell-to-cell communication.

This review seeks to shed more light on these unconventional and novel regulators of hematopoietic development and to present a broader picture of developmental and post-natal hematopoiesis.

## The Classical Regulators of Hematopoiesis

**Fig. 1:**
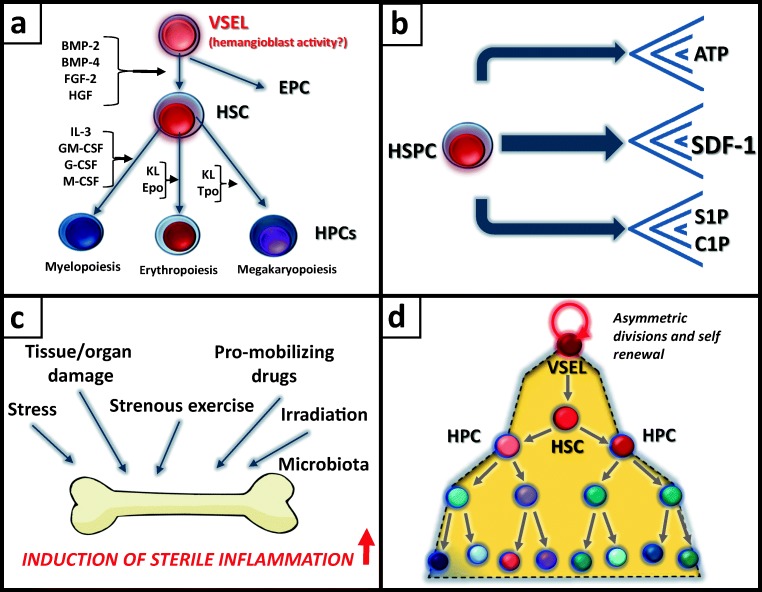
**Current topics in the development of hematopoietic stem cells. a** An open question is the phenotype of the most primitive hematopoietic stem cells (pre-HSCs) that give rise to HSCs and HPCs and perhaps other stem cells in BM microenvironment (EPCs, MSCs). It has been hypothesized that VSELs possess hemangioblast activity and are able to differentiate to both HSCs and EPCs. The most important peptide based factors operating at different levels of HSPCs specification into the various hematopoietic lineages are indicated. **b** The chemoattractants for HSPCs that have been identified so far are shown. In addition to SDF-1, some non-peptide-based factors, including ATP, S1P, and C1P, play an important role in both mobilization and homing of HSPCs. **c** Bone marrow is a tissue highly responsive to cues derived from damaged organs, including from stress, strenuous exercise, irradiation, pro-mobilizing agents, and gut microbiota. All these cues activate a state of “sterile inflammation” in BM microenvironment. **d** The current problems with expansion of HSCs are related to the problem that asymmetric division is needed to maintain the most primitive HSCs in an expanded pool of cells. VSELs seem to be an ideal starting population for this purpose, and it is worthwhile to explore them further and optimize their ex vivo expansion for this purpose

The most attention has been paid to classical hematopoietic growth factors and cytokines, which are employed in clonogenic assays in vitro to study colony formation by hematopoietic progenitor cells (HPCs) at different levels of their specification and maturation into hematopoietic lineages [4]. These progenitors are described as colony-forming units for mixed lineages (CFU-Mix), burst-forming units and colony-forming units for the erythroid lineage (BFU-E and BFU-E, respectively), burst-forming units and colony-forming units for the megakaryocytic lineage (BFU-Meg and CFU-Meg, respectively) as well as colony-forming units for granulocytes–monocytes (CFU-GM), granulocytes (CFU-G), monocytes (CFU-M), eosinophils (CFU-Eos), and basophils (CFU-Baso). These colonies are stimulated by combinations of (KL, Epo, Tpo, IL-3, GM-CSF, G-CSF, M-CSF. IL-4, and IL-5, Fig. 1a). More primitive HSPCs, such as those giving rise to cobblestone area-forming units or long-term repopulating hematopoietic stem cells (LT-HSCs), are potentially supported by BM-derived fibroblasts [4].

In this review we will not discuss these classical potent regulators, which are already treated in excellent publications [29–31], and instead focus mainly on other unconventional modulators of hematopoietic development.

## Other Peptide- and Non-peptide-Based Regulators of Hematopoiesis

Several factors have been described that regulate or co-regulate in a synergistic way the very early stages of embryonic hematopoiesis, including the specification of mesodermal precursors (pre-HSCs) and/or putative common endothelial-hematopoietic progenitor cells (hemangioblasts?) into HSCs [32, 33]. The most important are bone morphogenetic protein 2 and 4 (BMP-2 and BMP-4), basic fibroblast growth factor (bFGF), hepatocyte growth factor (HGF), and vascular endothelial factor (VEGF) [4].

Below we will address some other non-conventional regulators of hematopoietic development.

### Pituitary Hormones

Evidence has accumulated that several pituitary hormones, including follicle-stimulating hormone (FSH), luteinizing hormone (LH), prolactin (PRL), and growth hormone (GH), directly stimulate hematopoietic development by interacting with specific receptors expressed on the surface of HSCs [5, 6]. Functional receptors for these hormones have been described, not only on murine and human HSCs but also on more primitive populations of very small embryonic-like stem cells (VSELs), which are postulated to be the most primitive stem cells in postnatal BM and endowed with the potential to be specified into cells from different germ layers, including hematopoiesis [33, 36, 37]. In vivo injection of these hormones into mice expands the number of VSELs and HSCs in BM. Similalry, it has been reported that FSH may accelerate hematopoietic recovery in 5-FU-treated mice [38]. Moreover, the hematopoiesis-promoting effects of PRL [35] and LH [34] have been recently demonstrated also by other investigators.

### Gonadal Sex Hormones

The beneficial effects of steroid-backbone sex hormones on hematopoiesis is well documented for androgens, which have been employed for several years in the clinic as stimulators of hematopoiesis in cases of BM aplasia [8]. It has also been proposed that estrogens compensate for the loss of blood during menstruation in females. Overall, estrogens promote the cell cycle activity of HSPCs and induce their specification into megakaryocyte-erythroid progenitors, as seen for example during pregnancy [7]. Hematopoiesis in vivo is also stimulated in mice after the injection of progesterone. Interestingly, expression of pituitary and gonadal sex hormone receptors has been detected in murine and human VSELs and HSCs, which suggests a common developmental origin of these cells from migrating primordial germ cells (PGCs) [36, 37]. The potential developmental link between PGCs, VSELs, and HSCs is discussed later in this review.

### Other Hormones

In addition to gonadal sex hormones, other steroid-backbone hormones, such as glucocorticoids, are known for their positive effect on erythropoiesis and megakaryopoiesis [39]. They also indirectly affect leukocytosis by increasing the number of circulating neutrophils due to an accelerated release from BM and a reduced migration from the circulation. At the same time glucocorticoids reduce the number of circulating lymphocytes, monocytes, eosinophils, and basophils. The last stages of erythroid specification at the level of CFU-E progenitors are co-regulated by insulin-like growth factor 1 (IGF-1, also known as somatomedin C), which is secreted mainly from the liver into the circulation in response to GH stimulation [39, 40].

In zebrafish, an important role in the early stages of hematopoiesis has been documented for the extracellular nucleoside (EXN) adenosine [41]. The role of the entire family of EXNs in regulating trafficking of HSPCs and hematopoietic development will be discussed later in this review.

## The Developmental Origin of HSCs, and Is there a Link to Primordial Germ Cells (PGCs)?

The responsiveness of HSPCs to germ line-associated pituitary and gonadal sex hormones raises an important question: How are these cells responsive to these regulators? This important question can be answered by the fact that the first stem cells that become specified in the earliest stages of embryogenesis in the epiblast of the post-implantation blastocyst are primordial germ cells (PGCs) [36, 37, 42]. The epiblast is a precursor of the entire embryo proper, and PGCs are precursors of gametes, which pass genetic information, encoded in parental DNA and mitochondria, to the next generation. These cells, endowed with developmental totipotency, become specified in the proximal part of the epiblast and, shortly after specification, leave the embryo proper and migrate for a short period of time to the extra-embryonic mesoderm at the bottom of the yolk sac, where they begin to amplify, make a turn, and again enter the embryo proper through the primitive streak [42]. While continuing to be amplified in number, the PGCs migrate toward the genital ridges, where they settle and initiate gametogenesis. On their migratory route through the embryo proper toward the genital ridges they cross the part of the embryo called the aorta–gonado–mesonephros (AGM) region. In the wall of the dorsal aorta within this region the first definitive HSCs can be identified [31, 42].

Interestingly, the developmental route of PGCs during embryogenesis overlaps with the emergence of the first primitive HSCs in time and space—first in the so-called blood islands at the bottom of the yolk sac and later with the emergence of definitive HSCs in the AGM region of the developing embryo proper [31, 42]. Furthermore, both PGCs and HSCs are highly migratory stem cells, and it is very likely that some of the PGCs, while migrating in the extra-embryonic mesoderm, give rise as proposed to VSELs [36, 42] and VSEL-derived hemangioblasts [36, 37, 43, 44], which are precursors for both primitive HSCs and endothelial progenitor cells (EPCs). We envision that, while they migrate in the embryo proper through the AGM region towards the genital ridges, some of these cells become specified into definitive HSCs, which are detectable in the hemangiogenic endothelium of the dorsal aorta [37]. Other independent group that since many years studies migration of PGCs has also proposed this possibility recently [42]. Nevertheless, direct evidence is needed to demonstrate that VSEL can give rise to both HSC and EPC and thus comply with definition of hemangioblast. Such experiments are currently performed in our laboratory.

The developmental link between PGCs, VSELs, and HSCs may explain why VSELs and HSCs are responsive to several pituitary gonadotrophins and gonadal sex hormones and share certain molecular markers typical of germ development, such as the Sall4 germ line-specific transcription factor [37]. On the other hand, some germ line-derived cells (e.g., teratocarcinoma or gonadal cancer cells) express the erythropoietin receptor, which is known to be expressed by hemangioblasts and cells from the erythroid lineage [45].

## Modulators of Developmental and Post-Development Trafficking of HSPCs

HSCs are restless travelers [46–53]. After being specified as discussed above in the blood islands at the bottom of the yolk sac and later emerging in the hematogenic endothelium of the dorsal aorta in the AGM region, they migrate to the fetal liver, which becomes a major hematopoietic organ in the second trimester of gestation, both in mice and in humans. Subsequently, at the beginning of the third trimester of gestation they migrate from the fetal liver to the hematopoietic microenvironment of BM. Later in adult life, a small number of HSPCs circulates in peripheral blood and follows a circadian rhythm, in which the peak in their numbers occurs in the early morning hours and the nadir late at night [19.20,25,53].

HSPCs, in contrast to more mature hematopoietic and lymphopoietic cells (e.g., granulocytes, monocytes, or lymphocytes), respond by chemotaxis to a limited number of factors [47]. So far, besides the α-chemokine stromal-derived factor (SDF-1), which from a historical point of view is a “classical” chemoattractant for HSPCs, two bioactive phosphosphingolipids (S1P and C1P) [12–14] and the extracellular nucleotide ATP [15–17] have been described. These factors play a role not only in trafficking of adult HSPCs, as seen during mobilization and homing/engraftment, but most likely also during the developmental migration of HSPCs.

### Stromal-Derived Factor 1 (SDF-1)

SDF-1 is a well-known chemoattractant for fetal liver-residing HSPCs, which express its specific receptor, CXCR4, and by the end of the second trimester of gestation the SDF-1–CXCR4 axis relocates these cells to the hematopoietic microenvironment in developing bones [10, 11]. However, it is still not known which chemoattractant is crucial for attracting HSPCs from the anatomical sites where hematopoiesis is initiated (blood islands in the yolk sac and the dorsal aorta) into the fetal liver, as SDF-1-KO and CXCR-KO mice die in utero and have a normal number of myeloid HSPCs in their fetal liver [10, 11]. It is possible that S1P, C1P, or ATP here play an important role [47, 54]. Moreover, when studying SDF-1 as a chemoattractant one has to consider that, in experimental chemotaxis assays in vitro, this chemokine is employed at supra-physiological concentrations [47]. Therefore, more attention should be focused on S1P, C1P and ATP chemotactic activities, which are very potent chemoattractants for these cells at tissue-expressed (physiological) levels (Fig. 1b). These non-peptide-based factors are discussed below.

### Bioactive Phosphosphingolipids (S1P and C1P)

Initially, S1P was identified as an important regulator of the trafficking of lymphoid cells [55]. However, in the meantime evidence has accumulated that HSPCs strongly respond to chemotactic gradients of S1P and C1P. An increase in these gradients in peripheral blood (PB) during the mobilization of HSPCs directs their egress from BM [12–14, 56]. Similarly, an increase in S1P and C1P level in BM after conditioning for transplantation plays a supportive role in SDF-1-mediated homing and engraftment of these cells. Functional receptors for S1P (S1P_R1–5_) are expressed on HSCs, and S1P_R1_ and S1P_R3_ seem to be most important in regulating the trafficking of HSPCs [57]. In contrast to S1P receptors, the receptor/s for C1P have not yet been identified. However, the inhibition of C1P-mediated chemotaxis by pertussis toxin suggest that, like S1P, these receptors are G_i_ protein-coupled, seven-transmembrane-spanning receptors [47].

### Extracellular Nucleotides

Adenosine triphosphate (ATP) is known to be involved in energy transfer inside cells. However, ATP is also released from activated and stressed cells and in the extracellular compartment becomes an important signaling molecule involved in purinergic signaling [15, 58, 59]. In the extracellular space ATP is hydrolyzed to ADP, AMP, and finally adenosine, which are all mediators of purinergic signaling. The purinergic signaling receptor family is comprised of seven P2X (P2X1–7), eight P2Y (P2Y1, 2, 4, 6, 11, 12, 13, and 14), and four P1 (A1, A2a, A2b, and A3) receptors expressed on the surface of hematopoietic cells, including HSPCs. The P1 and P2Y receptors are G protein-coupled receptors, while the P2X receptors are ligand-gated ion channel receptors [59]. While ATP stimulates P2X receptors, adenosine stimulates P1 receptors. In addition to ATP, P2Y receptors are stimulated by other nucleotides, including ADP, UTP, UDP, and UDP-glucose [58, 59]. ATP is an important modulator of HSPC trafficking; however, exposure to high levels of ATP may lead to apoptosis of these cells. The involvement of adenosine in the early stages of hematopoiesis in zebrafish was mentioned above [41]. Our results indicate that, while ATP promotes migration of HSPCs, adenosine is an important inhibitor of migration [16, 17].

### Adrenergic Signaling

It has also been demonstrated that mobilization of HSPCs is regulated by β-adrenergic signaling [19, 20]. Bone marrow tissue is innervated and sympathetic synapses identified in proximity to BM stromal cells. Adrenergic signaling has been implicated in regulating the circadian release of HSPCs from BM into PB. Nevertheless, one must consider that this effect could be co-regulated by purinergic signaling, as ATP is released together with adrenalin into the synaptic space, and purinergic receptors are highly expressed on neural cells as well as in synapses [58]. However, this intriguing concept requires further experimental support.

## Sterile Inflammation of the Bone Marrow Microenvironment

Bone marrow tissue is a dynamic structure that is highly responsive to several non-inflammatory cues related to stress, tissue or organ injuries, strenuous exercise, gut microbiota-derived signals, certain drugs, and radio-therapy (Fig. 1c) [17, 47]. Moreover, this dynamic environment of BM tissue coordinates signals derived from activation of the complement cascade (ComC), the coagulation cascade (Coa), the fibrinolytic cascade (FibC), mediators of the neural system, and the redox state of the organism [50, 51]. In all these situations danger-associated molecular pattern molecules (DAMPs) are released from the affected cells and activate i) cell-surface Toll-like receptors and ii) intracellular nucleotide-binding oligomerization domain (NOD)-like receptors (e.g., Nlrp) [49]. As demonstrated recently, the intracellular Nlrp3 receptor, known as the Nlrp3 inflammasome, is crucial for regulating the trafficking of normal HSPCs under both steady-state and stress conditions, including pharmacological mobilization [60, 61]. The Nlrp3 inflammasome is activated by the ATP–P2X7 receptor interaction, which leads to the release of DAMPs (high molecular group box 1 [HMGB1] and S1009a) and certain cytokines (IL-1β and IL-18) [62, 63], which activate the ComC and CoaC. These mutual interactions provide a basis for better understanding how innate immunity regulates hematopoiesis.

In a state of infection, these receptors may also be activated by pathogen-associated molecular patter molecules (PAMPs). By contrast, under steady-state conditions physiological commensal bacteria, such as those in the gut, may also indirectly affect bone marrow by secreted liposaccharide (LPS). LPS activates toll-like receptor 4 (TLR4) and primes the Nlrp3 inflammasome for synthesis of IL-1β and IL-18 [62, 63]. Depletion of the gut microbiota by antibiotics impairs hematopoiesis and the trafficking of HSPCs [21, 22]. Mice exposed to antibiotics that eliminate gut microbiota are poor mobilizers of HSPCs. This effect is most likely mediated by a decrease in the priming effect of LPS, via the TLR4 receptor, on the basic tonus of the Nlrp3 inflammasome and synthesis of IL-1β and IL-18 [62, 63].

In addition to these interactions, a new player in cell-to-cell communication has emerged: extracellular microvesicles (ExMVs) [27, 28]. ExMVs secreted from mesenchymal cells have been demonstrated to protect HSPCs from irradiation injury in the BM microenvironment. The biological effects of ExMVs are mediated by their cargo, which includes mRNA, miRNA, proteins, and bioactive lipids that inhibit apoptosis and promote proliferation of HSPCs [64]. ExMVs have been demonstrated to facilitate engraftment of HSPCs as well as directly stimulate HSC proliferation. In allogeneic hematopoietic transplantation they may also ameliorate graft-versus-host disease (GvHD) [65]. The role of ExMVs in hematopoiesis is an exciting and still largely unwritten chapter in stem cell biology.

## Strategies to Accelerate Hematopoietic Reconstitution after Transplantation of HSPCs

Successful and rapid engraftment of HSPCs after transplantation directly impacts the survival rate of patients. It can be achieved by transplantation of a high number of HSPCs directly obtained from a donor or their effective ex vivo expansion before administration to the patient [47]. Therefore, on the one hand, optimization of stem cell mobilization protocols is crucial for harvesting a high number of HSPCs for transplantation. Besides G-CSF or AMD3100, other potent co-stimulators of mobilization have been proposed for testing in the clinic including N-terminal truncated form of the human CXC chemokine GRO-beta [66], inhibitor of adhesion molecule very late antigen-4 (VLA-4) inhibitor [67] and dipeptidylpeptidase-4/CD26 (DPP4/CD26) truncation product the neurotransmitter neuropeptide Y (NPY) [68]. The armamentarium of such potential pro-mobilizing agents has increased, and recent research has also demonstrated that mobilization is enhanced by the addition of i) drugs that increase the level of S1P in PB [69], ii) inhibitors of extracellular adenosine synthesis in the bone marrow microenvironment [16, 17], or iii) inhibitors of heme oxygenase 1 (HO-1) [70, 71] and inducible nitric oxide synthetize [72], intracellular enzymes which inhibit the mobilization process.

On the other hand, in the case of poor mobilizers or poor donors of BM cells, an important option is ex vivo expansion of harvested HSPCs. Here, however, is a problem, as most of the expansion protocols using cocktails of growth factors expand mostly HPCs at the expense of HSCs [47]. Nevertheless, a significant breakthrough in expansion strategies has been achieved by using, in addition to cocktails of growth factors or cytokines, small molecules such as StemRegenin (SR1), an aryl hydrocarbon receptor (AhR) antagonist [73]; nicotinamide (NAM), an amide form of vitamin B3 [74]; or a pyrimidoindole derivative (UM171) [75]. What is intriguing, both UM171 and NMA were also successfully employed to expand ex vivo VSELs into HSCs [76, 77].

Interestingly, in all reported expansion approaches using NMA and UM171 small-molecule modifiers, the HSCs for expansion were isolated using paramagnetic beads against CD34 or CD133 antigens expressed by these cells [74, 75, 78]. Therefore, from a suspension of BM, mPB, or UCB hematopoietic cells, paramagnetic beads pull down both large and small CD34 and/or CD133 cells. Since small VSELs express both CD34 and CD133 antigen, they are co-isolated with larger HSCs. Taking into consideration the kinetics of expansion, we hypothesize that cells expanded using these protocols were in fact pre-HSCs or VSELs [47, 76, 77]. This question needs further clarification, and the potential positioning of VSELs in the hierarchy of stem cells during ex vivo expansion is depicted in Fig. 1d. With this hierarchy in mind, VSELs seem to be the most suitable cells for expansion and asymmetric division and would likely maintain the pool of the most primitive pre-HSCs during this procedure.

## Future Directions in HSPC Research and Applications

Despite significant progress in understanding hematopoiesis, there are several issues that still have to be addressed. First, as mentioned above, it is still not clear what are the most primitive stem cells in BM that give rise to HSCs. Evidence accumulated that this are VSELs [43, 47, 79, 80]. Interestingly, since we expect from most primitive stem cells to undergo assymetric divisions, recently published data demonstrated that VSELs indeed undergo assymetric divisions. Accordingly, CD45^−^NUMB^−^ VSELs differentiate into CD45^+^NUMB^+^ HSCs [80]. The NUMB protein is evolutionary conserved and plays a role in the determination of cell fates during development. NUMB localizes to one side of the mother cell and it is distributed selectively to one daughter cell only during assymetric division. This division allows a daughter cell that contains NUMB protein to acquire a different fate than the other daughter cell after division [81].

Moreover, it is still an open question whether adult tissues contain a hemangioblast, a stem cell that can give a rise to HSCs and endothelial progenitor cells (EPCs) [32, 33]. This possibility has several pros and cons, and more research is needed to give a final answer. In this context it is very likely that the VSELs reported in adult postnatal tissues can be enriched for a population of putative hemangioblasts [33]. Next, since the number of HSPC donors is limited, it is important to identify new sources of these cells for clinical applications. It has been proposed that HSPCs could be generated from embryonic stem cells (ESCs) or induced pluripotent stem cells (iPSCs) [82]. However, the problem with these cells is that, aside from the risk of teratoma formation and rejection by the recipient, they have significant genomic instability, and so far no engraftable HSPCs have been derived from ESCs or iPSCs [82, 83]. Here again, adult tissue-derived VSELs, which have been successfully expanded into the hematopoietic lineage and can be engrafted in immunodeficient mice [82, 84], could become a valuable option for solving this problem. In addition to SR1, NAM, and UM171, other potent expansion-promoting molecules have been developed. Again these new compounds (e.g., BIO or GSK3β inhibitor, trichostatin A, NR1010) should be tested for expansion and hematopoietic specification of VSELs.

Since HSPCs from mPB are a valuable source of cells for transplantation, more efficient mobilization strategies for cases of poor mobilizers should be developed. Moreover, it is important to identify genetic markers for poor mobilizers. As previously demonstrated, one of the crucial pathways involved in mobilization is activation of purinergic signaling. In this pathway, ATP is released from stressed cells by pannexin 1 channels and binds to P2X7 purinergic receptors to activate the Nlrp3 inflammasome [59–61]. Our recent patient results revealed that ~60% of patients that were poor HSPC mobilizers (*n* = 20) displayed the pannexin 1 polymorphism SNP5 (Rs3020015) T/C. By contrast, this polymorphism was observed in only 1 out of 26 good-mobilizer patients (~4%) (manuscript in preparation). Furthermore, as recently reported, the presence of the Gln460Arg SNP polymorphism within the purinergic P2X7 receptor gene in HSPCs, which is always co-inherited with Ala348Thr to form the gain-of-function haplotype 4, resulted in a significant increase in CD34^+^ HSPC mobilization [85]. This again supports the important role of purinergic signaling in this process [15–17].

Finally, an important task is to improve the seeding efficiency of transplanted HSPCs to BM hematopoietic niches. To facilitate this process, ex vivo priming strategies have been proposed in which the cells to be grafted are selected according to their responsiveness to a homing gradient of SDF-1 [47]. This process could be enhanced by processing HSPCs for transplant in hypoxic conditions [23, 24] or exposing them to short term mild heating in 39 °C [86], overexpressing in these cells HOXB4 [87] or pharmacological upregulation of HIF-1a by caffeic acid phenentyl ester [88]. Moreover, some factors have been identified, including anti-microbial cationic peptides that are part of the innate immunity response, such as (i) the complement cascade cleavage fragment C3a, (ii) cathelicidin (LL-37), and iii) β2-defensin, which are induced by activation of the ComC in BM stroma during conditioning for transplantation of HSPCs [89]. A short exposure of HSPCs to these factors conditions them for transplantation before injection into recipients and accelerates homing and engraftment [90]. This is explained by increased incorporation of CXCR4 homing receptor for SDF-1 into membrane lipid rafts [91]. Interestingly, a similar effect is achieved by exposure of HSPCs to prostaglandin E2 (PGE2) as explained due to upregulation of CXCR4 receptor on HSCs [91]. Other potential strategies facilitating homing of HSPCs were the subject of a recent review in *Stem Cell Reviews and Reports* [92].

Moreover, since intracellular HO-1 in HSPCs negatively affects their migration, homing and engraftment, exposure of cells in the graft to small-molecule inhibitors of HO-1 could enhance hematopoietic reconstitution. This has been demonstrated in an in vivo model in mice [93].

Many of these promising strategies mentioned above were obtained in experimental animal models and await validation in well controlled clinical studies.

## Conclusions

Hematopoiesis and development of HSPCs are intriguing and still hold many secrets. Bold new concepts, validated by experiments, are needed to unleash their full regenerative potential. Paradigms in science change with time and, as Albert Einstein stated, “*Blind belief in authority is the greatest enemy of truth*” and as his countryman Max Planck said “*One rule is important in science—only courageous people win*”. In hematopoiesis there are still many doors to be opened in order to understand this fascinating cell system, and courageous young investigators are needed. In pursuing scientific truth it is also important to be critical and to keep in mind another famous saying from Albert Einstein “*Anyone who has never made a mistake has never tried anything new*” and one from Maria Sklodowska-Curie “*I was thought that the way of progress is neither swift or easy*”.
